# Supplementary material for: One Step In Situ Co-Crystallization of Dapsone and Polyethylene Glycols during Fluidized Bed Granulation
Source: Pharmaceutics. 2023 Sep 16;15(9):2330. doi: 10.3390/pharmaceutics15092330 (PMC10535358; doi:10.3390/pharmaceutics15092330)
Supplement: Supplementary file 1 [file pharmaceutics-15-02330-s001.zip › pharmaceutics-2580812-supplementary.pdf]

Supplementary Materials

One Step In Situ Co-Crystallization of Dapsone and Polyethylene Glycols during Fluidized Bed Granulation

Shizhe Shao [1,2], David Bonner [1], Brendan Twamley [3], Abhishek Singh [4] and Anne Marie Healy [1,2,*]

[1] School of Pharmacy and Pharmaceutical Sciences, Trinity College Dublin, D02 PN40 Dublin, Ireland; shaos@tcd.ie (S.S.); bonnerd@tcd.ie (D.B.)
[2] SSPC, The Science Foundation Ireland Research Centre for Pharmaceuticals, School of Pharmacy and Pharmaceutical Sciences, Trinity College Dublin, D02 PN40 Dublin, Ireland
[3] School of Chemistry, Trinity College Dublin, D02 PN40 Dublin, Ireland; twamleyb@tcd.ie
[4] Janssen Pharmaceutica NV, 2340 Beerse, Belgium; asing195@its.jnj.com
* Correspondence: healyam@tcd.ie; Tel.: +353-1-8961444

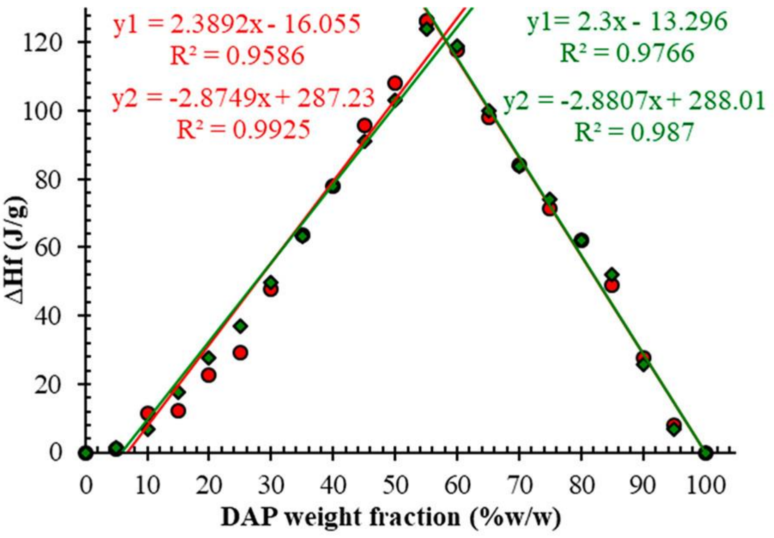

		Slop	Intercept
Experiment-1	Y1	2.3892	-16.055
	Y2	-2.8749	287.23
	**Intersection point X1**	**57.6**	
Experiment-2	Y1	2.3	-13.296
	Y2	-2.8807	288.01
	**Intersection point X2**	**57.3**	

Figure S1. The plot of weight proportion of DAP and PEG1450 mixture against the melting enthalpy of its mixture. Reproduced with permission from Praveen Chappa, Crystal Growth & Design, published by Chappa et al., 2018 [14].

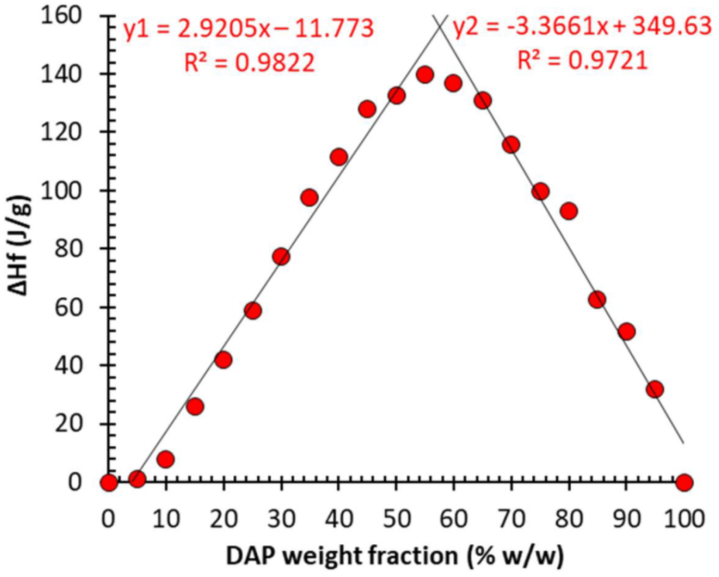

	Slop	Intercept
y1	2.9205	-11.773
y2	-3.3661	349.63
**Intersection point X1**		**57.5**

Figure S2. The plot of weight proportion of DAP and PEG6000 mixture against the melting enthalpy of its mixture. Reproduced with permission from Praveen Chappa, Crystal Growth & Design, published by Chappa et al., 2018 [14].

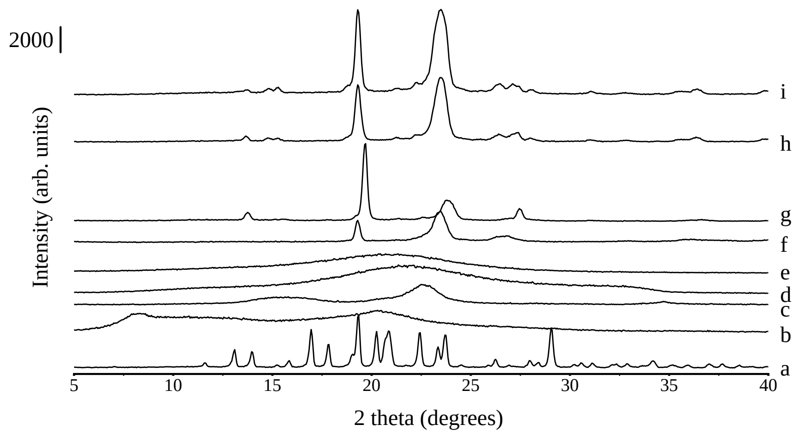

Figure S3. XRD patterns of each raw material. a) DAP (form III), b) HPMC, c) MCC, d) PEG 400, e) PEG 600, f) PEG 1000, g) PEG 1500, h) PEG 4000, i) PEG 6000

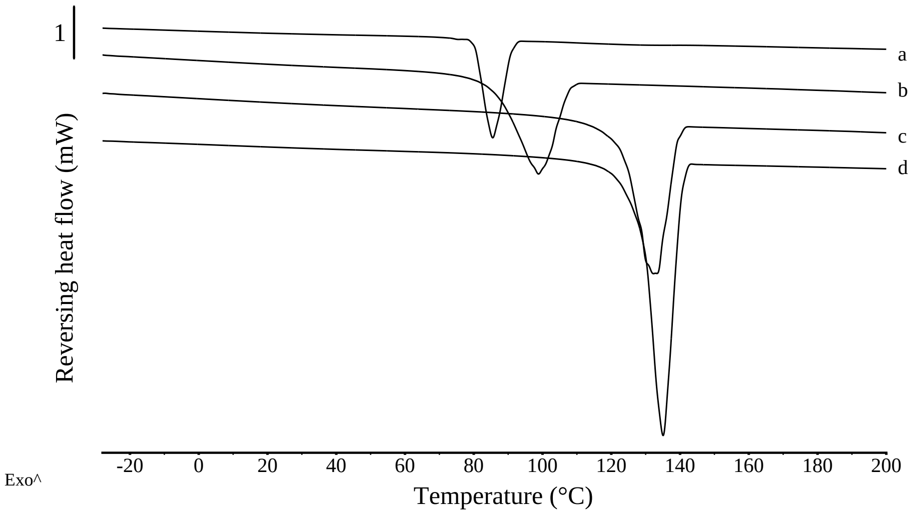

Figure S4. Reversing heat flow of (a) F1, (b) F5, (c) F9, (d) F10 granules.

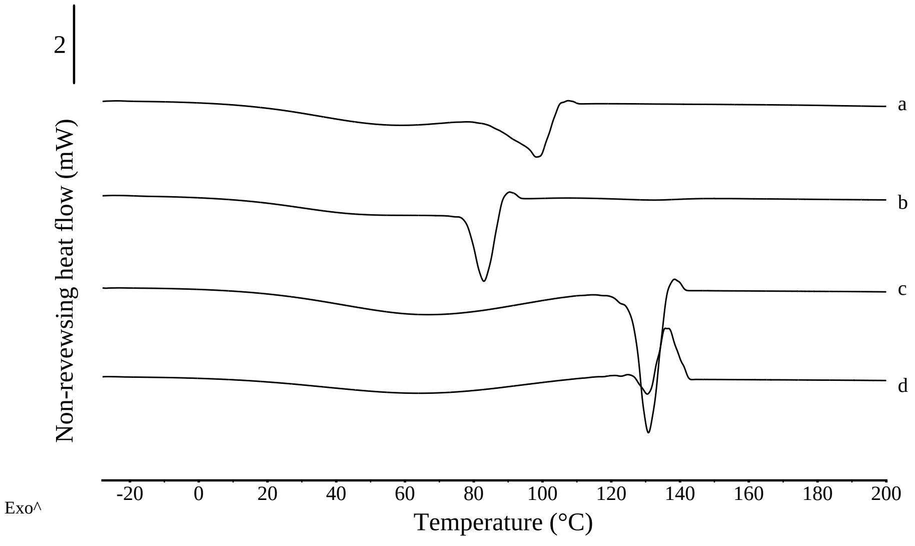

Figure S5. Non-reversing heat flow of (a) F1, (b) F5, (c) F9, (d) F10 granules.

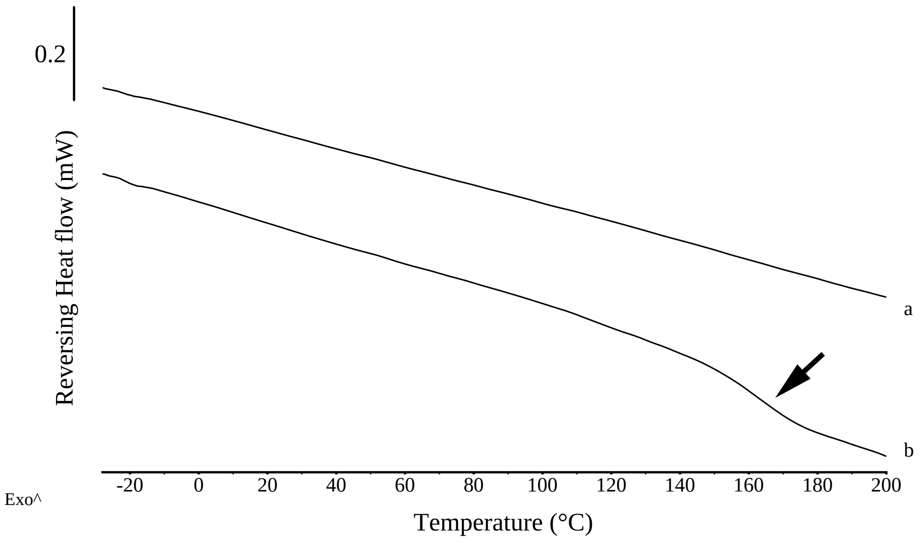

Figure S6. Reversing heat flow of (a) MCC and (b) HPMC raw materials. The arrow represents the glass transition of HPMC.

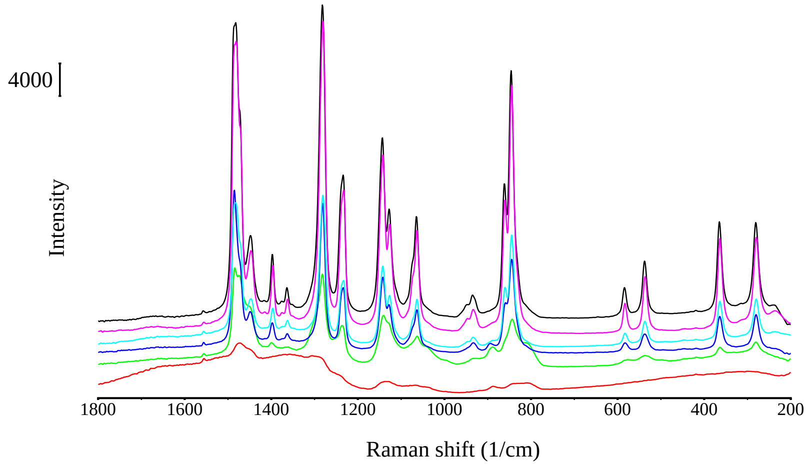

Figure S7. Raman spectra of PEG 400 (red line), PEG 600 (green line), PEG 1000 (blue line), PEG 1500 (cyan line), PEG 4000 (purple line), and PEG 6000 (black line).

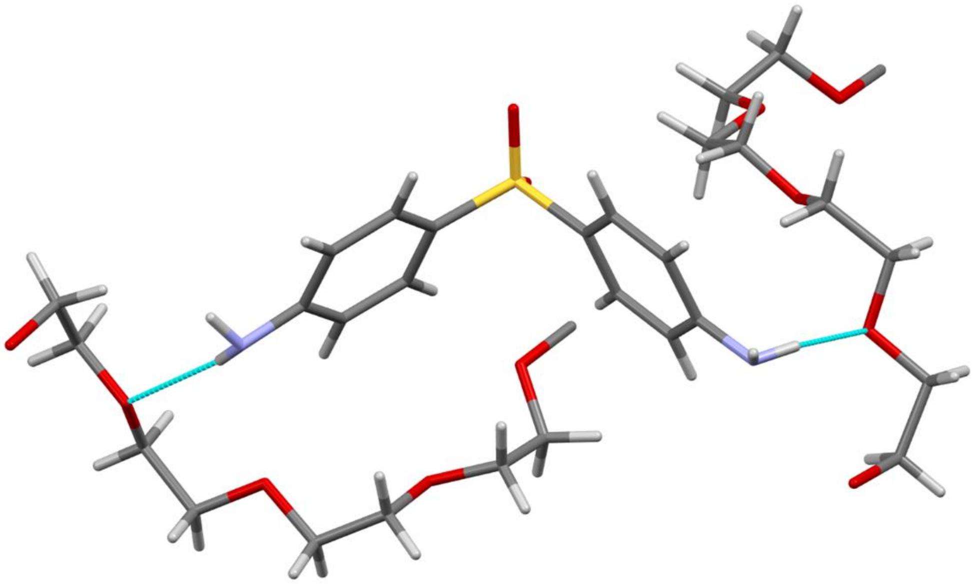

Figure S8. 3D diagram of the molecular interaction of DAP-PEG 600 co-crystal derived from the reported CCDC identifier number 1868167 [14].

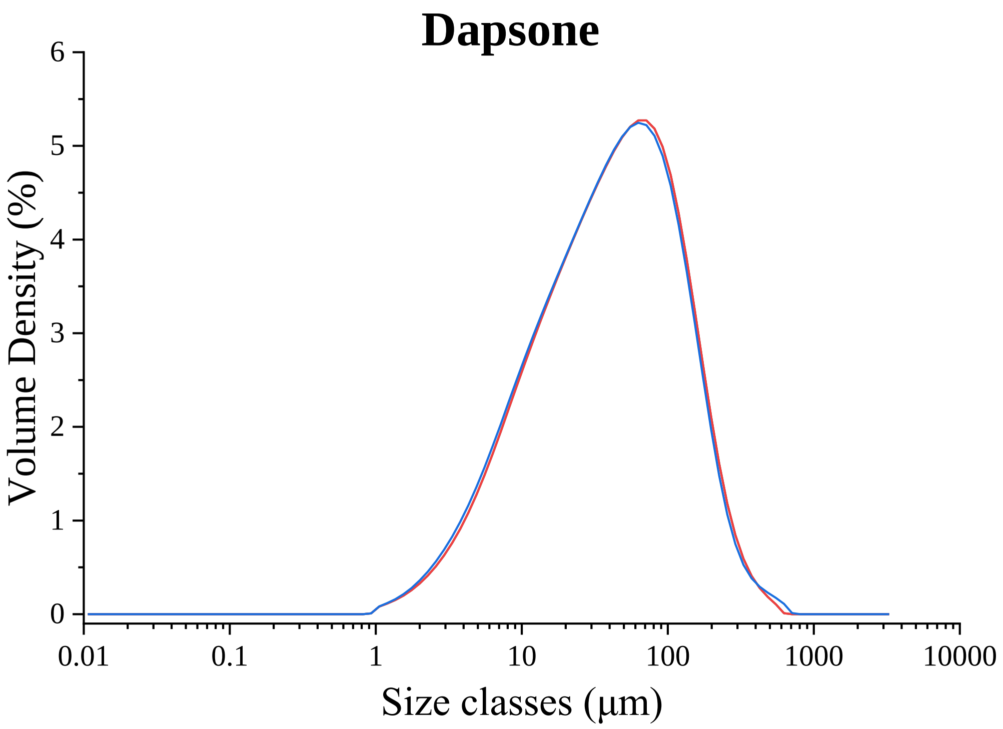

Figure S9. Particle size distribution of DAP raw material at 3 bar (n=3). Each line represents one analysis
